# Clonal and subclonal TP53 molecular impairment is associated with prognosis and progression in multiple myeloma

**DOI:** 10.1038/s41408-022-00610-y

**Published:** 2022-01-26

**Authors:** M. Martello, A. Poletti, E. Borsi, V. Solli, L. Dozza, S. Barbato, E. Zamagni, P. Tacchetti, L. Pantani, K. Mancuso, I. Vigliotta, I. Rizzello, S. Rocchi, S. Armuzzi, N. Testoni, G. Marzocchi, G. Martinelli, M. Cavo, C. Terragna

**Affiliations:** 1 IRCCS Azienda Ospedaliero-Universitaria di Bologna, Seràgnoli Institute of Hematology, Bologna, Italy; 2 Department of Experimental, Diagnostic and Specialty Medicine - University of Bologna, Bologna, Italy; 3 Department of Medical Oncology, Istituto Scientifico Romagnolo per lo Studio e la Cura dei Tumori (IRST) IRCCS, Meldola, Italy

**Keywords:** Risk factors, Myeloma, Cancer genomics, Myeloma, Translational research

## Abstract

Aberrations on *TP53*, either as deletions of chromosome 17p (del17p) or mutations, are associated with poor outcome in multiple myeloma (MM), but conventional detection methods currently in use underestimate their incidence, hindering an optimal risk assessment and prognostication of MM patients. We have investigated the altered status of *TP53* gene by SNPs array and sequencing techniques in a homogenous cohort of 143 newly diagnosed MM patients, evaluated both at diagnosis and at first relapse: single-hit on *TP53* gene, either deletion or mutation, detected both at clonal and sub-clonal level, had a minor effect on outcomes. Conversely, the coexistence of both *TP53* deletion and mutation, which defined the so-called double-hit patients, was associated with the worst clinical outcome (PFS: HR 3.34 [95% CI: 1.37–8.12] *p* = 0.008; OS: HR 3.47 [95% CI: 1.18–10.24] *p* = 0.02). Moreover, the analysis of longitudinal samples pointed out that *TP53* allelic status might increase during the disease course. Notably, the acquisition of *TP53* alterations at relapse dramatically worsened the clinical course of patients. Overall, our analyses showed these techniques to be highly sensitive to identify TP53 aberrations at sub-clonal level, emphasizing the poor prognosis associated with double-hit MM patients.

## Introduction

In most human cancers, impairment of p53 tumor suppressor protein is a driver event, which confers a survival advantage to tumor cells [1, 2]. Clonal aberrations of *TP53* gene—either hemizygous deletion of the short arm of chromosome 17 (del17p) or mutations—adversely affect the prognosis of multiple myeloma (MM) patients, regardless of therapy [3–5]. Among p53 abnormalities, del(17p) and/or monosomy 17 are listed as the worst prognostic factors, being del(17p) also associated with resistance to chemotherapy and increased risk of disease spread outside the bone marrow [6–8]. Since the presence of del(17p) is one of the cytogenetic variables, along with t(4;14) and/or t(14;16) contributing to define stage 3 disease according to the revised International Staging System (R-ISS) [9–11], routine assessment of clonal p53 status is strongly recommended. Fluorescent in situ hybridization (FISH), the commonest technique used to reveal del(17p), fails to detect, or otherwise underestimates, focal copy number (CN) deletions and/or point mutations affecting *TP53*, especially when sub-clonal [12], thus preventing an accurate risk stratification of MM. This issue is of particular relevance when the TP53 loss of function occurs through a bi-allelic event [13], a finding currently associated with the worst prognosis.

The prognostic value of sub-clonal p53 gene deletion has been recently highlighted in a large cohort of patients, even though just a limited set of selected *TP53* exons has been evaluated by multiplex ligation-dependent probe amplification (MLPA) [14]. Additionally, a subsequent study established a threshold of 0.55 of Cancer Cell Fraction (CCF) as a cut-off capable of discriminating patients with different risks of progression [15]. However, despite the large consensus regarding the prognostic value of p53 alterations, especially in case of its bi-allelic inactivation [13, 15, 16], the identification of the most effective methods to assess p53 impairment, as well as the optimal cut-off levels for these determinations, remain controversial.

Herein, we explored the *TP53* full-gene genomic status in a cohort of newly diagnosed MM patients and in a subgroup for whom longitudinally collected samples were available, in order to determine, by means of single-nucleotide polymorphisms (SNPs) array and targeted sequencing, the prognostic significance of *TP53* CN and mutational events, as well as their evolution along the disease course.

## Methods

### Patients

Hundred and forty-three NDMM patients for whom bone marrow samples taken at diagnosis were available for *TP53* analysis were included into this study. Their median follow-up was 72 months, range: 4–192; interquartile range [IQR]: 46.7–119.1 Of these patients, 53 with paired bone marrow samples at diagnosis and at relapse were analysed for *TP53* status. All patients were treated upfront with bortezomib-based regimens, 98 of them within clinical studies, either the GIMEMA-MMY-3006 trial (*n* = 45 patients) (ClinicalTrials.gov number: NCT01134484) or the EMNO2/HO95 trial (*n* = 53 patients) (ClinicalTrials.gov number: NCT01208766). 100 out of 143 patients received consolidation therapy with high-dose melphalan (HDM) followed by autologous stem-cell transplantation (ASCT), whereas 43 patients received bortezomib-based regimens as intensification therapy. All patients provided written informed consents for biological studies.

Baseline clinical characteristics were representative of a MM population; however, since β2-microglobulin levels were slightly unbalanced among the three subgroups, the analyses have been adjusted for ISS, whose impact on patient outcome has been already demonstrated (Table 1 in SI), to avoid any potential bias and to properly correlate the clinical outcome with cytogenetic abnormalities.

### Sample processing

Bone marrow (BM) aspirates were obtained during standard diagnostic procedures. Mononuclear BM cells were obtained by Ficoll-Hypaque density gradient centrifugation. An immunomagnetic beads-based strategy (MACS system, Miltenyi Biotec, Auburn, CA) was employed to isolate CD138+ plasma cells. The purity of positively selected plasma cells was assessed by flow cytometry using a conventional antibody panel. Total genomic DNA was isolated using Maxwell® 16 LEV Blood DNA kit (Promega, Madison, WI) and quality/quantity checked by Nanodrop. Western blotting analysis on CD138+ cell derived from two MM patients, was performed as previously published [17].

### Genome wide SNP array profiles and CNAs detection

SNP array profile experiments were carried out according to the manufacturer’s protocols (Cytoscan HD Genome-wide Human GeneChip, Affymetrix, Santa Clara, CA). Raw CEL files were processed by a pipeline including Rawcopy [18] and ASCAT [19] to compute purity-corrected CN data. Specific log *ratio* thresholds were set to correctly define *TP53* ploidy [20]. Del17p, t(4;14), t(14;20), and t(14;16) were also evaluated by FISH analysis in a subgroup of patients (Vysis LSI Probes, Abbott Molecular). SNP CEL files are available for free download at http://www.ncbi.nlm.nih.gov (GEO, Gene Expression Omnibus), series accession number GSE69000 [21]. Purity solutions with a low confidence were manually reviewed, and custom R scripts were used in order to obtain gene-level copy number calls for *TP53* locus. The genomic segments profiles were generated using Raw copy R package and PSCBS algorithm. The significance threshold for segmentation was set at 10^−7^. The copy number thresholds for single copy gain and single copy loss were set at 2.1 and 1.9, respectively. The copy number thresholds for two or more copy gain and homozygous loss were set at 3.4 and 0.6, respectively [22]. According to the purity of ASCAT computed samples, Log2 *ratio* signals were subsequently converted to CN values and a CCF was defined for each alteration that spans from 0 to 100%.

### *TP53* targeted deep sequencing and variant calling

*TP53* gene was sequenced by a probe-based targeted sequence panel of 25 genes, among which the whole exonic regions of TP53 was included (Sophia genetics). In each run, a TP53 mutated cell line (OPM-2) was included as positive control. A total of 22 samples were re-sequenced in order to test the reproducibility intra and inter-run. Based on their availability, 42-matched normal samples (e.g. buccal swab) were sequenced. Somatic variants, included for analysis, passed NextSeq Reporter quality filter and met laboratory-defined,thresholds of >250× read depth and >5% variant allele fraction (VAF) [23, 24]. Data on two selected TP53 variants were further validated through a droplet digital PCR assay (ddPCR), both at DNA and RNA level. Sequencing data were aligned to GRCh37-hg19 genome assembly and subjected to pre-processing steps for variant discovery following GATK best practices [25]. For variant calling analysis, a concordance of three different tools was employed: Mutect2 (Broad Institute), Sophia DDM (proprietary software), and Shearwater: we considered a variant only if it was called by at least two out of these three tools. Data pre-processing for variant discovery was carried out independently for Mutect2/Shearwater using GATK4 best practices, while data pre-processing for Sophia DDM was part of the Sophia proprietary analysis pipeline. Tool-specific variant filtering steps were applied on the different lists of variants: 1) Mutect2 variants were filtered using all available filtering criteria provided by FilterMutectCalls tool, and only variants with all PASS flags were considered; 2) Shearwater variants were considered only with a Quality score >30; 3) Sophia DDM variants labeled with a PASS flag from the software were considered. All the filtering steps were manually reviewed with Integrative Genomics Viewer (IGV) to evaluate the filtering performance. Regarding the biological significance filtering, in order to differentiate pathogenic from variant of uncertain significance (VUS) or benign variants, we set up an algorithm capable of assigning a pathogenicity label to the considered variants, using all the currently available information from public databases and in-silico prediction tools, obtained by a variant annotation step with ANNOVAR. Briefly, pathogenic variants were labeled as such, if: 1) they caused a biological loss of function (e.g., missense, nonsense, and frameshift), or 2) were reported as pathogenic in clinical databases (e.g., CLINVAR, COSMIC) and had a strong evidence of pathogenicity from in-silico prediction tools. After filtering, we focused only on the pathogenic detected variants by excluding common variants in human population (freq. >1%) and retaining only variants with a confirmed evidence of pathogenicity in comprehensive clinical databases (i.e., COSMIC, CLINVAR).

### Clinical and statistical analyses

All the analyses were conducted using R language and environment for statistical computing (R Foundation for Statistical Computing, Vienna, Austria). 0.05 was considered as the limit for the statistical significance of *p*-value and all variables objected of inference were reported together with their 95% confidence intervals (CI). A time-dependent receiving operating characteristic (ROC) curve analysis [26] was performed in order to measure the best progression-free survival (PFS) estimate at different time-points (from 12 to 96 months) of TP53 CCF. PFS was calculated from the start of therapy to the first progression or death. Overall survival (OS) considered death as event and was calculated from the same landmark. Second PFS and PFS2 were defined as the time to 2nd progression or death: PFS2 was calculated from start of therapy, while 2nd PFS measurements started from the date of first progression. Survival curves were drawn following the Kaplan–Meier method. Semi-parametric Cox regression analysis was adopted to calculate hazard ratios (HR) between predefined possible prognostic groups, including all the cytogenetic alterations (del1p, amp1q, del17p, del13q, and translocations t(4;14)). Multivariable Cox regression analysis was performed to identify the abnormalities independently affecting the prognosis, considering in the final reported model only the variables that resulted statistically significative.

## Results

### Patients carrying clonal and sub-clonal 17p deletion, and particularly double-hit events, displayed poor prognosis, and higher probability of second relapses

To assess the molecular status of TP53, both at copy number and at mutational levels, SNPs array and high-resolution targeted sequencing were performed. In order to define the optimal prognostic cutoff levels for *TP53* deletion calling and the lowest TP53 CCF value predicting for clinical outcomes, a ROC curve approach was employed. To this aim, each 0.05-progressively reduced *TP53* CCF value was tested, starting from CCF = 2, equivalent to the normal diploid CN. As expected, clonal deletions (CCF = >63%) allowed a confident and early prediction of PFS and OS events (12 months, AUC = 0.84) (Tables 2–3 in SI). However, we were able to show that the lowest acceptable *TP53* CCF limits still able to significantly predict PFS (96 months) and OS (72 months) were 9.56% and 11.32%, respectively (AUC = 0.62). We therefore defined 10% (as an approximation of both 9.56 and 11.32%) as the cut-off level for a *TP53* deletion call.

According to the established cut-offs, thirty-four patients carried *TP53* deletions (34/143 = 24%) and 12 patients *TP53* mutations (12/143 = 8%); among these, seven patients carried double-hits on *TP53* (7/143 = 5%), being affected either by homozygous deletions (2/7) or by deletion and mutation (5/7), whereas 97 patients carried wild-type (wt) *TP53*.

**Fig. 1:**
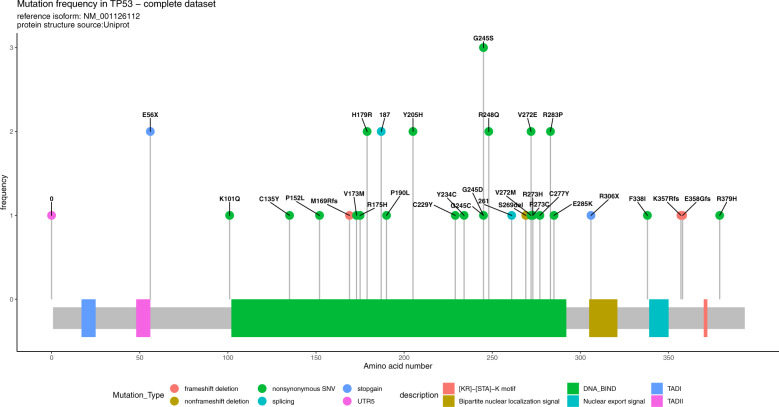
Lollipop plot representing TP53 number and frequency of mutations. Most of the SNVs are restricted to the DNA binding domain; however, other SNVs are detected in other flaking domains.

Mutations were mainly non-synonymous single-nucleotide variants (SNVs) and affected the DNA binding domain (Fig. 1).

**Fig. 2:**
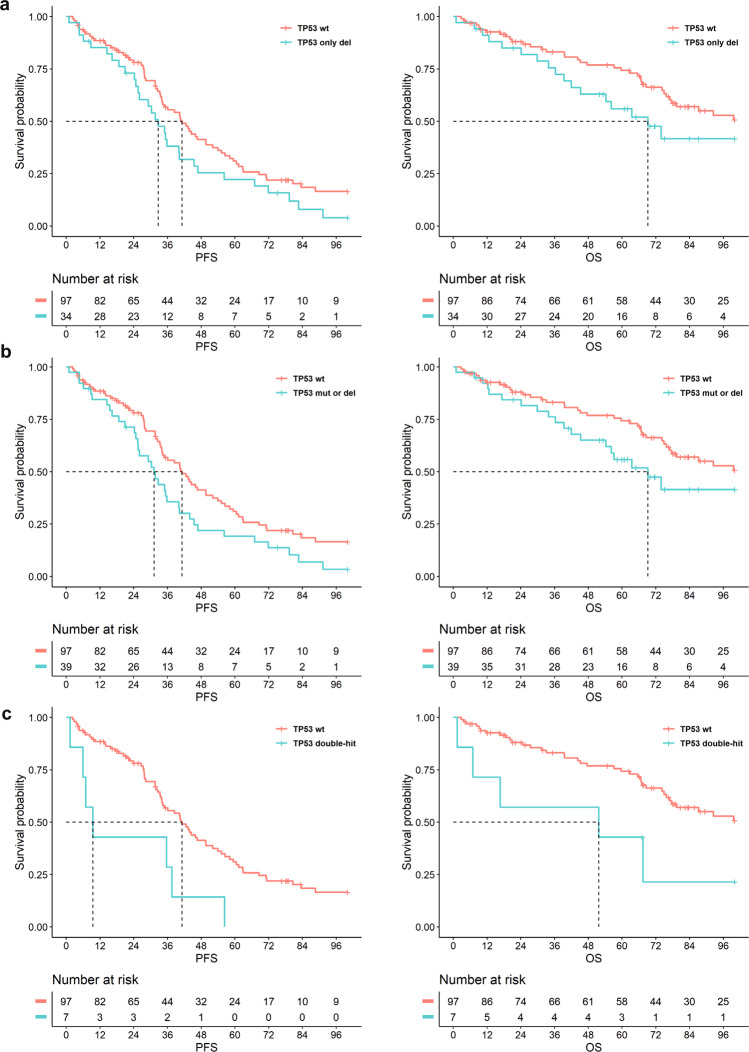
Clinical impact of clonal and subclonal TP53 aberrations at diagnosis. Effect on PFS and OS of **a** TP53 deletion as a single-hit (cut-off ≥10%) as detected by SNP array (PFS: *p* = 0.06, HR 1.57, 95% CI: 1.02–2.43; OS: *p* = 0.05, HR 1.79, 95% CI: 1.01–3.2); **b** TP53 deletion or mutation (VAF ≥ 5%) (PFS: *p* = 0.02, HR 1.63, 95% CI: 1.07–2.48; OS: *p* = 0.05, HR 1.82, 95% CI: 1.02–3.26); **c** coexistence of both deletions and mutations on TP53, which defined the so-called double-hit patients (PFS: *p* = 0.01, HR 3.34, 95% CI: 1.37–8.12; OS: *p* = 0.02, HR 3.47, 95% CI: 1.18–10.24).

As previously reported, the clinical impact of p53 impairment might be heterogeneous according to its allelic status [13]. Our results demonstrated that patients with *TP53* deletion, both clonal and sub-clonal, displayed a substantial outcome worsening, as compared to patients with wt *TP53* (Fig. 2a: PFS median months: 32.7 vs. 41.2, respectively, HR 1.57, 95% CI: 1.02–2.43, *p* = 0.06; OS median months: 69.2 vs. not reached [nr], HR 1.79, 95% CI: 1.01–3.2, *p* = 0.05), even though only a trend was observed in term of PFS.

Overall, the presence of a single genomic hit on *TP53*, either a deletion or a mutation (as observed in 39 patients) significantly impacted patients’ survival (Fig. 2b: PFS median months: 31.2 vs. 41.2 respectively, HR 1.63, 95% CI: 1.07–2.48, *p* = 0.02; OS median months: 69.2 months vs. nr, HR 1.82, 95% CI: 1.02–3.26, *p* = 0.05). More importantly, the simultaneous occurrence of *TP53* deletion and mutation, or of *TP53* bi-allelic deletion, severely affected the clinical outcome of patients (Fig. 2c: PFS median months: 9.5 vs. 41.2, respectively: HR 3.34, 95% CI: 1.37–8.12, *p* = 0.008; OS median: 51.7 vs. nr, HR 3.47, 95% CI: 1.18–10.24, *p* = 0.02) (see also Fig. 1 in SI). On this side, a double event ultimately resulted in a complete inactivation of p53 protein (Fig. 2 in SI, pt2), while a single *TP53* copy loss ensured intact p53 full-length protein, as well as that of phosphorylated p53 protein (Fig. 2 in SI, pt1).

In a multivariable prediction model, only the statistically significant variables were included. As a result, *TP53* double-hit events influenced independently and more heavily patient outcomes, with respect to FISH-detected del(17p), both in terms of PFS and OS (Tab. 4 in SI).

**Fig. 3:**
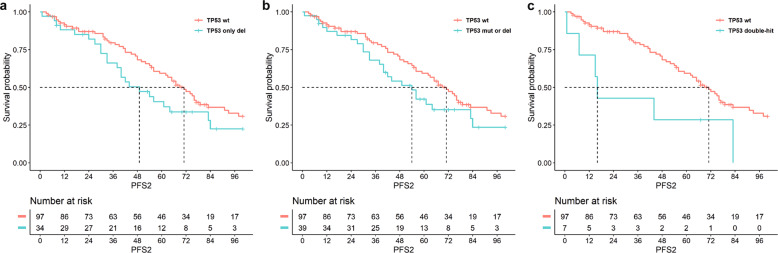
Risk of second relapses resulted higher in patients carrying at diagnosis TP53 deletion or mutation, including double-hit events, as compared to patients carrying wild-type TP53. Effect on PFS2 of **a** TP53 deletion as a single-hit (cut-off ≥10%) as detected by SNP array (*p* = 0.04, HR 1.71, 95% CI: 1.03–2.84); **b** TP53 deletion or mutation (VAF ≥ 5%) (*p* = 0.05, HR 1.62, 95% CI: 0.99–2.66); **c** coexistence of both deletions and mutations on TP53 (*p* = 0.02, HR 3.10, 95% CI: 1.18–8.17).

Finally, patients carrying either single-hit (deletion or mutation) or double-hit events on *TP53* at diagnosis had higher risk to experience second relapses, as compared to patients carrying wild-type *TP53* (Fig. 3: PFS2: (a) 34 pts with TP53-del vs. 97 pts with wt-TP53, median months survival: 54 vs. 71, respectively, HR 1.71, 95% CI: 1.03–2.84, *p* = 0.03; (b) 39 pts with single-hit (deletion or mutation) on TP53 vs. 97 pts with wt-TP53, median months survival: 49 vs. 71, respectively: HR 1.62, 95% CI: 0.99–2,66, *p* = 0.05; (c) 7 pts with double-hit on TP53 vs. 97 pts with wt-TP53, median months survival: 16 vs. 71, respectively: HR 3.10, 95% CI: 1.18–8.17, *p* = 0.02).

### *TP53* genomic state might evolve along the disease course, therefore its assessment is crucial both at diagnosis and at relapse

**Fig. 4:**
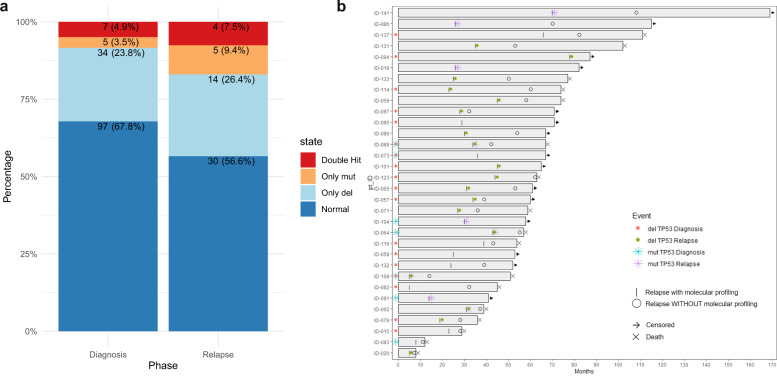
TP53 molecular status might change during the disease progression and impact in long-term outcomes. **a** Histogram representing the frequencies of TP53 aberrations across diagnosis and relapse. Overall, we observed an increase of the relative percentage of patients carrying TP53 aberrations at relapse ([45/143] 32% at diagnosis vs. [23/53] 44% at relapse; *p* < 0.05). **b** Swimmerplot representing how TP53 molecular status change across disease phases (e.g., diagnosis and relapse). Even though in most patients the TP53 status (either altered or not) remained stable along the disease course, 14/53 patients acquired either deletion (8/53), or mutations (4/53) or double-hit events (2/53) at relapse.

In order to verify if *TP53* molecular status might change between disease phases, 53 out of 143 patients were molecularly re-assessed at the time of disease progression. By analyzing these longitudinally collected samples, we observed an overall increase of frequency of patients carrying *TP53* aberrations at relapse ([45/143] 32% patients with a *TP53* deletion, mutation or both at diagnosis vs. [23/53] 44% patients with a *TP53* deletion, mutation or both at relapse; *p* < 0.05), showing an acquisition of either deletion (14/53), or mutations (5/53) or double-hit events (5/53) at relapse (Fig. 4). Interestingly, not only an increased number of genomic events on *TP53*, but also a *TP53* CCF raise was observed at relapse, as compared to diagnosis (median *TP53* CCF: 62.9% [range: 10–100%] vs. 82.4% [range: 25.5–100%] at diagnosis and relapse, respectively, *p* < 0.05).

**Fig. 5:**
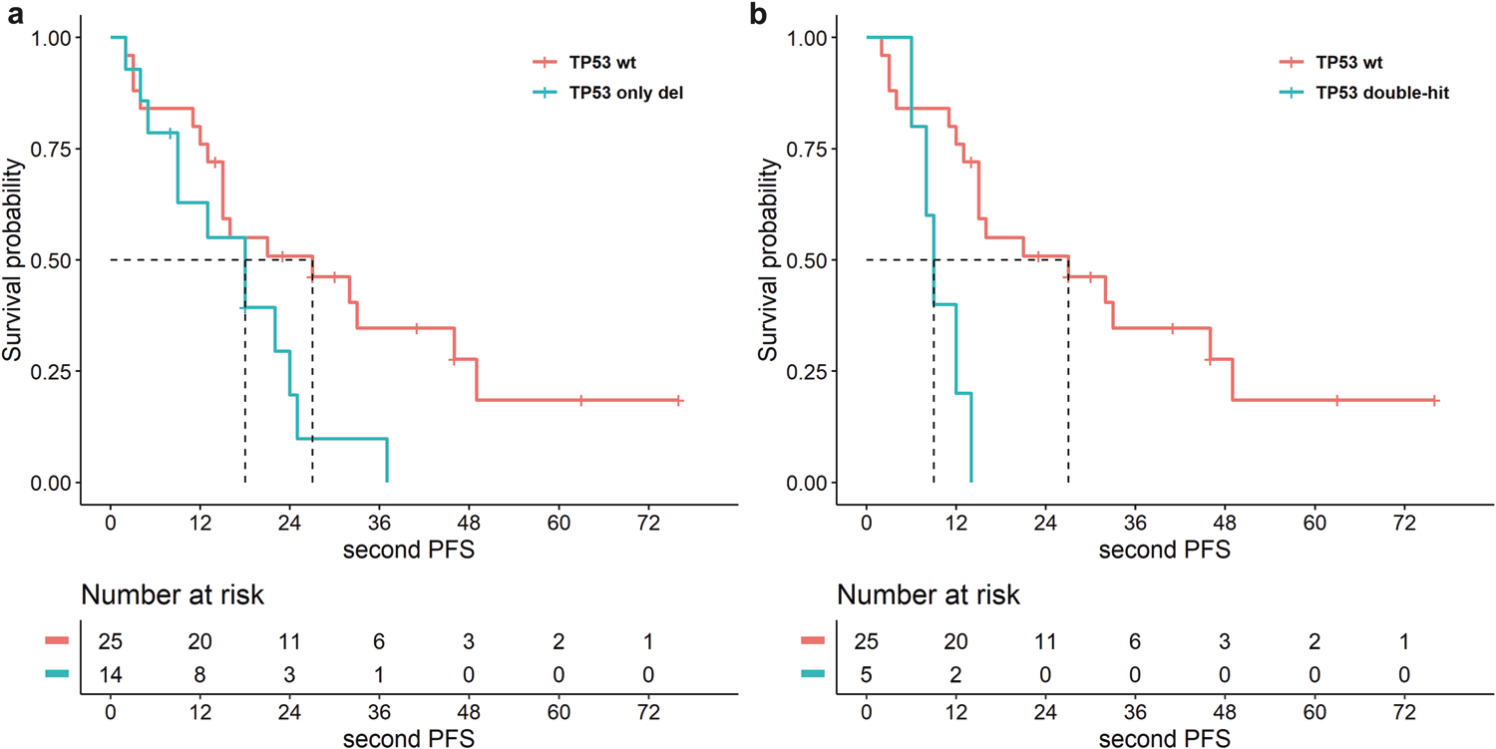
The acquisition of TP53 alterations at relapse dramatically threatened patients’ clinical course. Analysis of 2nd PFS of **a** patients with TP53 deletion (cut-off ≥10%) (*p* = 0.05, HR 2.39, 95% CI: 1.01–5.64); **b** coexistence of both deletions and mutations on TP53 (*p* = 0.02, HR 4.80, 95% CI: 1.27–18.13).

Among patients who reported an increased frequency/number of *TP53* mutations at relapse, a validation of two *TP53* variants in two distinct patients was performed by ddPCR (Fig. 3 in SI). In patient 1, the frequency of *TP53* var 166G>T (ex4) displayed an increase from 12 to 36.3%, confirmed both at DNA and at RNA levels. More strikingly, in patients 2, the frequency of *TP53* var 848G>C (ex8) showed a variation from 0.62 to 7.2%, thus still remaining sub-clonal, but even though detectable both at DNA and RNA levels. The acquisition of TP53 aberrations at relapse affected patients’ clinical course even worse than at diagnosis, as we demonstrated by the analysis of 2^nd^ PFS (Fig. 5: (a) 14 pts with TP53 del vs. 25 pts with wt TP53, 18 vs. 27 months: HR 2.39, 95% CI: 1.01–5.64, *p* = 0.04; (b) 5 pts with double-hit on TP53 vs. 25 pts with wt TP53, 9 vs. 27 months: HR 4.80, 95% CI: 1.27–18.13, *p* = 0.02).

## Discussion

Genomic events affecting the 17p chromosomal regions are quite common in cancer and are mostly related with the loss of p53 tumor suppressor function, ultimately impacting patients prognosis [1, 2]. Deletion of 17p also occurs in MM, although less rare than in other tumor types. In fact, it is rarely observed in the pre-clinical phases and only 8–10% of patients in the daily clinical practice have a FISH-detected del(17p) at the onset of the disease [3, 4]. Despite its relatively low incidence and the consideration of being a secondary event in the pathogenesis of MM [26], del(17p) remains one of the most clinically relevant chromosomal aberration, as its presence define a high-risk patient’s category in several approved risk scores [27, 28]. However, the identification of the best approach to assess p53 impairment and the cut-off levels for these determinations are still controversial. Here we have demonstrated the feasibility of high-resolution detection of TP53 aberrations, down to the sub-clonal level, performed at both diagnosis and relapse. Overall, our findings reinforce the idea that both deletions and mutations should be evaluated to correctly identify NDMM patients with poor prognosis, and that the evaluation should be repeated at disease recurrence.

Although the use of high-sensitive techniques, including both focal and sub-clonal genomic events, inevitably identified a rather high recurrence of *TP53* events (*TP53* deletions: 34/143 = 24% and *TP53* mutations: 12/143 = 8%), as compared to less performing techniques [3, 4], the ROC curve analysis supported the relevance of the detecting both clonal and sub-clonal *TP53* alterations. In fact, while clonal deletions (CCF > 63–100%) had an early impact on patient outcome, sub-clonal *TP53* alterations (CCF > 10–63%) also proved a strong, albeit delayed, clinical role, proving to be an equally crucial genomic event. On this basis, besides the identification of clonal *TP53* events, unequivocally relevant, we strongly support the inclusion of high-sensitive methods to detect sub-clonal events at diagnosis, since they have the potential to more accurately define patients at high risk of progression. Indeed, our results showed that both *TP53* CCF spanning from 100 to 10% and *TP53* mutations VAF lower than 5% at the time of disease onset, impacted patient’s survival, despite the sub-clonal nature of the lesion in certain patients.

More importantly, we showed that double-hit events on *TP53*, either homozygous deletion or deletion plus mutation, completely compromised p53 protein function, deeply worsening the clinical outcomes of patients and doubling the risk of second relapses. These data were further validated in a multivariable model, where double-hit events impacted the clinical outcome, independently from *TP53* deletion, as detected by the conventional approach which, however, leaves out the presence of possible mutations. The importance to assess both type of alterations (deletion and mutation) was further corroborated by results on p53 protein, showing that the single-hit of *TP53* was not sufficient to cause the p53 loss of function, completely abrogated when double-hit events occurred.

Collectively, these data highlight the importance of a wide molecular approach, aimed at defining *TP53* genomic status to proper assess the risk of disease in MM patients and, concomitantly, avoid the underestimation of *TP53* genomic aberrations.

In the last years, studies derived from large data repository have revealed that MM is characterized by the co-existence of heterogenous clones and sub-clones, being either suppressed or selected under the therapeutic selective pressure during the disease course, eventually defining a linear, neutral, or branched evolution. On this side, even though del(17p) is commonly reported as a clonal event, early detection of sub-clones carrying this aberration in newly diagnosed MM patients, along with longitudinal analysis of samples to assess TP53 molecular status both at diagnosis and at relapse, might represent a critical warning and should not be ignored.

Taken together, these data supported the relevance of *TP53* genomic status in NDMM, by accurately describing the CN and the mutational status of this gene, mainly aiming at the identification of *TP53* double-hit events. We demonstrated that both clonal and sub-clonal *TP53* aberrations significantly impaired clinical outcome of MM patients, particularly when *TP53* was totally compromised, as in case of double-hit events. Moreover, we suggest del17p 10% CCF threshold to be used both for risk assessment of patients enrolled in clinical trials and for diagnostic testing in NDMM. We also showed that the *TP53* molecular status has proven clinically meaningful even at relapse, supporting the role of a re-assessment of *TP53* molecular status at relapse. Finally, this study emphasized the superiority of highly sensitive molecular approaches, such as SNPs array and Next Generation Sequencing, over conventional methods. The combination of these techniques with the conventional use of FISH in clinical practice and in the diagnostic routine will improve a proper risk stratification and prognostication of MM patients.

## Supplementary information

TP53_SUPPINFO
