# Supplementary material for: Clonal and subclonal TP53 molecular impairment is associated with prognosis and progression in multiple myeloma
Source: Blood Cancer J. 2022 Jan 26;12(1):15. doi: 10.1038/s41408-022-00610-y (PMC8791929; doi:10.1038/s41408-022-00610-y)
Supplement: Supplementary file 1 — TP53_SUPPINFO [file 41408_2022_610_MOESM1_ESM.docx]

**SUPPLEMENTARY INFORMATION**

***Table 1 SI – Baseline clinical characteristics of the patients’ cohort included in the study stratified in three main subgroups according to the treatment (Daily Clinical Practice [DCP], GIMEMA-MMY-3006 and HOVON-EMN02).*** HB, haemoglobin; PLTs, platelets; PCs, plasma cells; Ca, calcium; ISS, International Staging System.

**
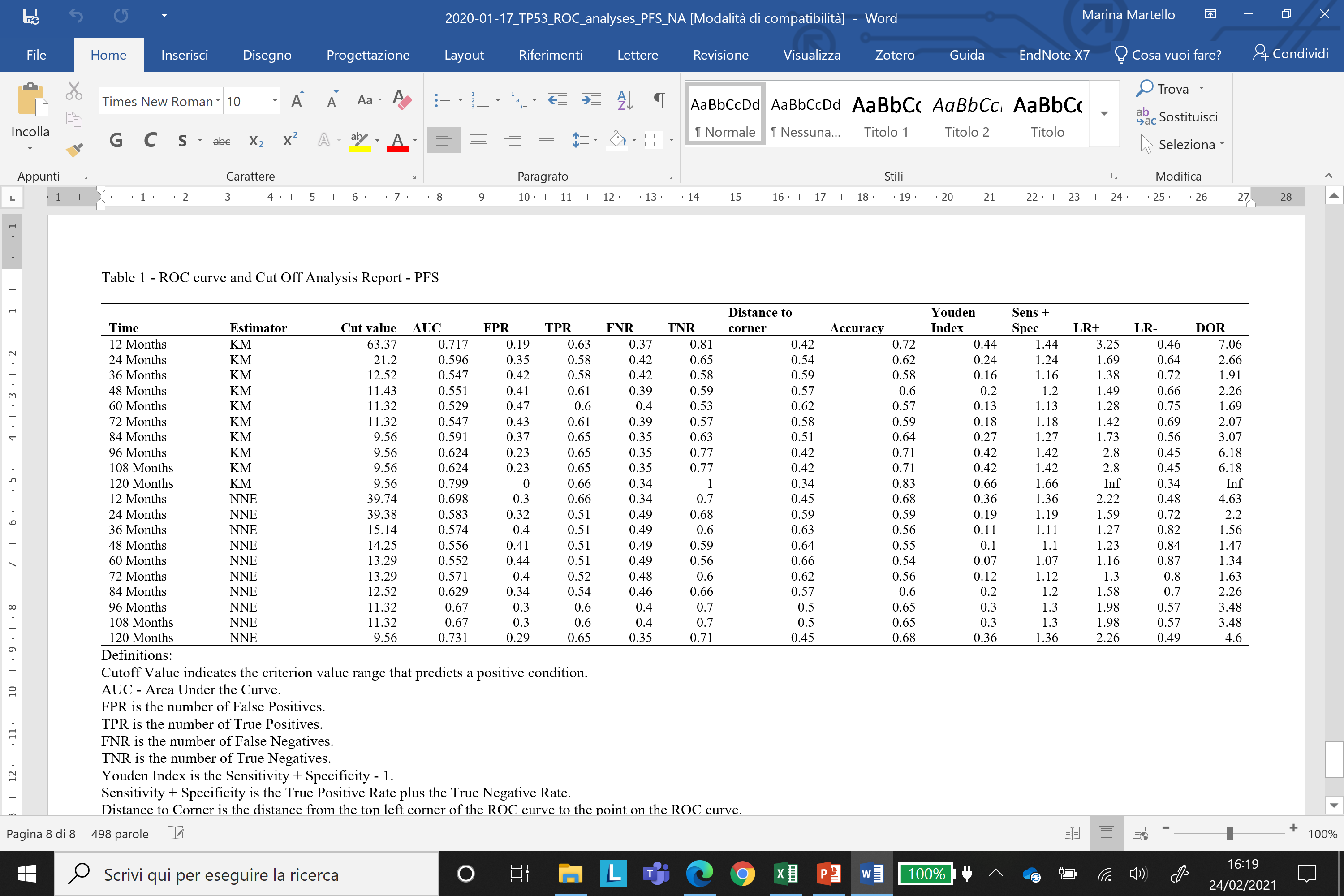
**

***Table 2 SI - ROC curve Analysis on Progression Free Survival (PFS) months.*** KM, Kaplan Meyer; AUC, Area Under Curve; FPR, False Positive Rate; TPR, True positive rate; FNR, False Negative Rate; TNR, True Negative Rate.

**
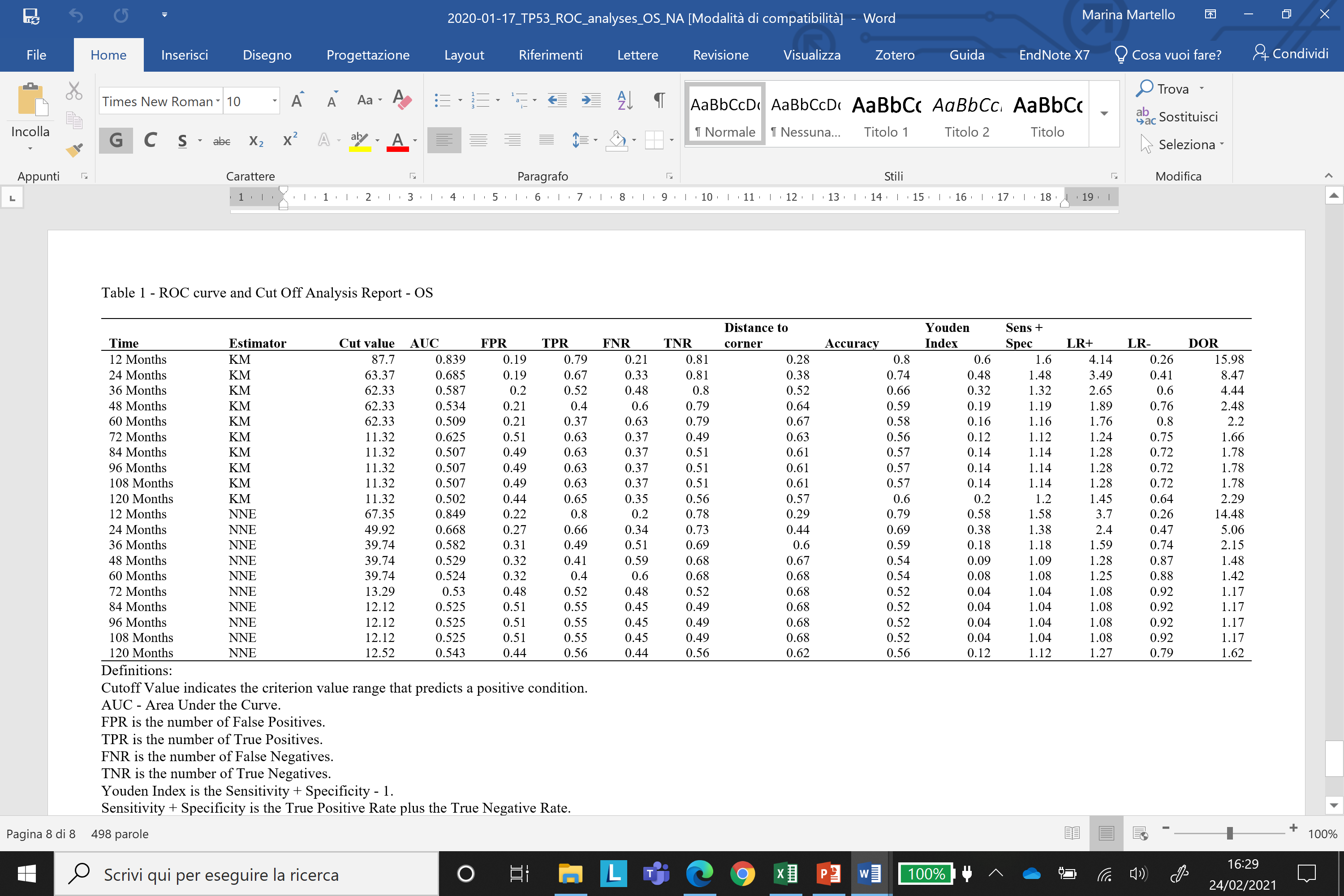
**

***Table 3 SI - ROC curve Analysis on Overall Survival (OS) months****.* KM, Kaplan Meyer; AUC, Area Under Curve; FPR, False Positive Rate; TPR, True positive rate; FNR, False Negative Rate; TNR, True Negative Rate.

**Figure 1 SI - Effect on PFS, OS and PFS2 of TP53 wt, TP53 single-hit and TP53 double-hit**


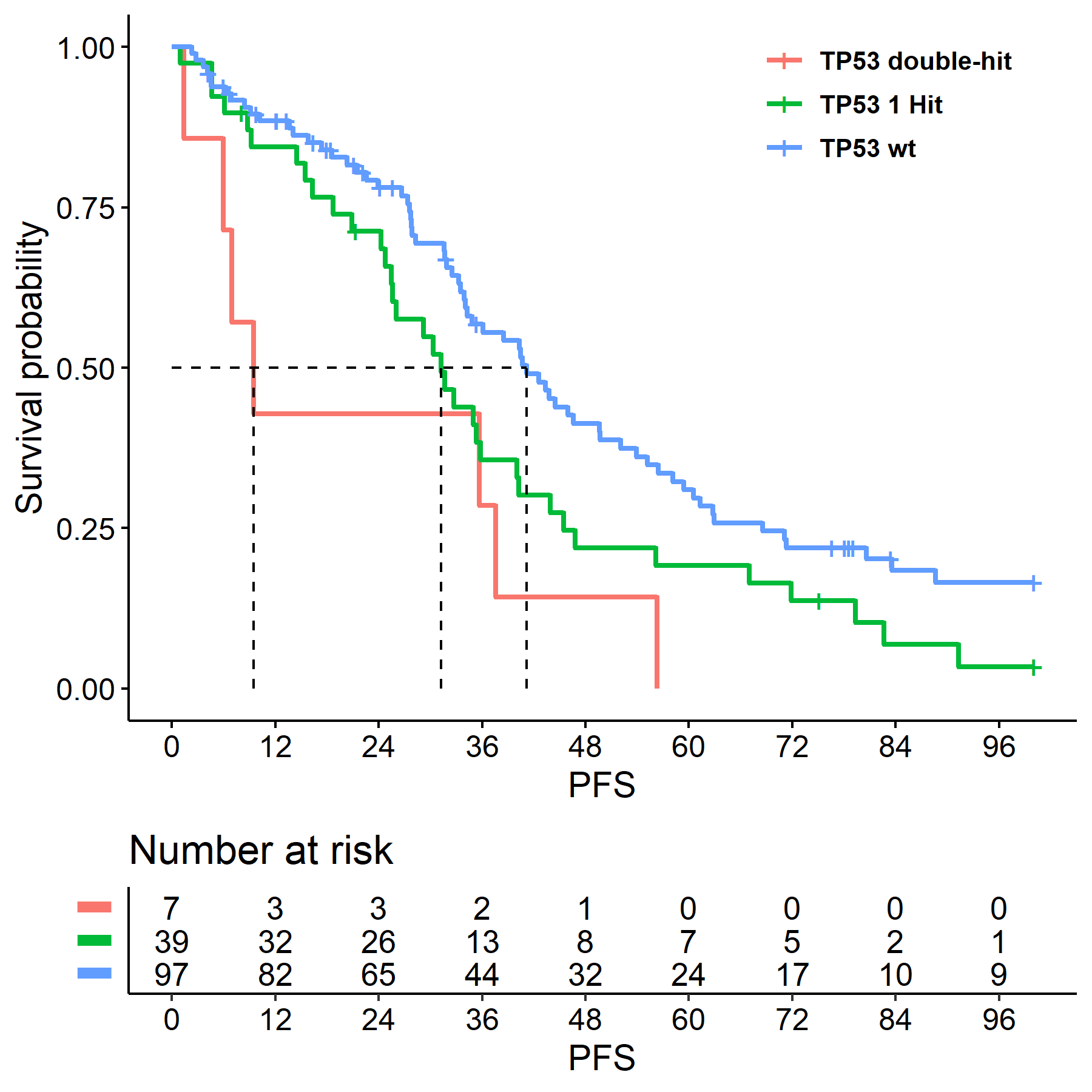

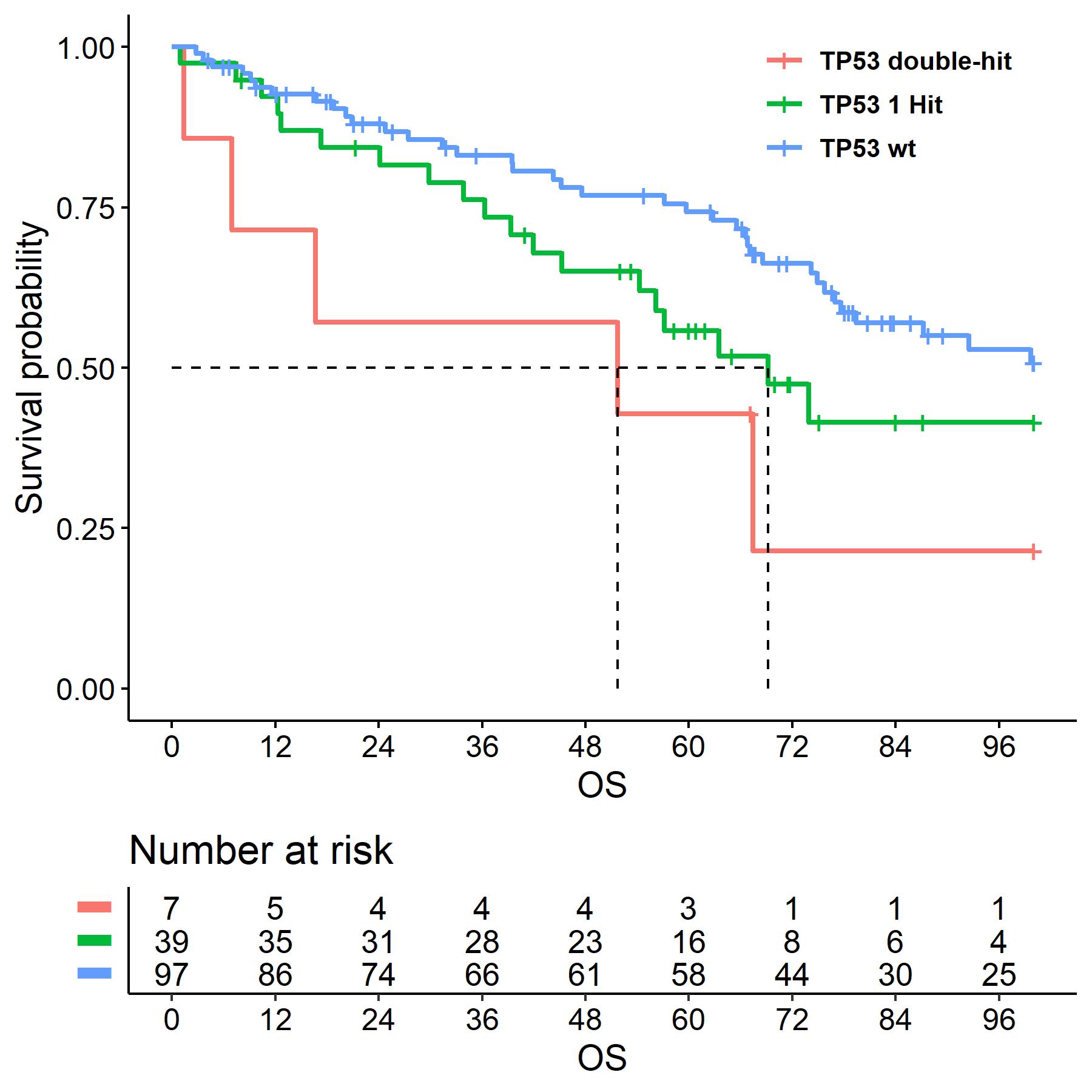

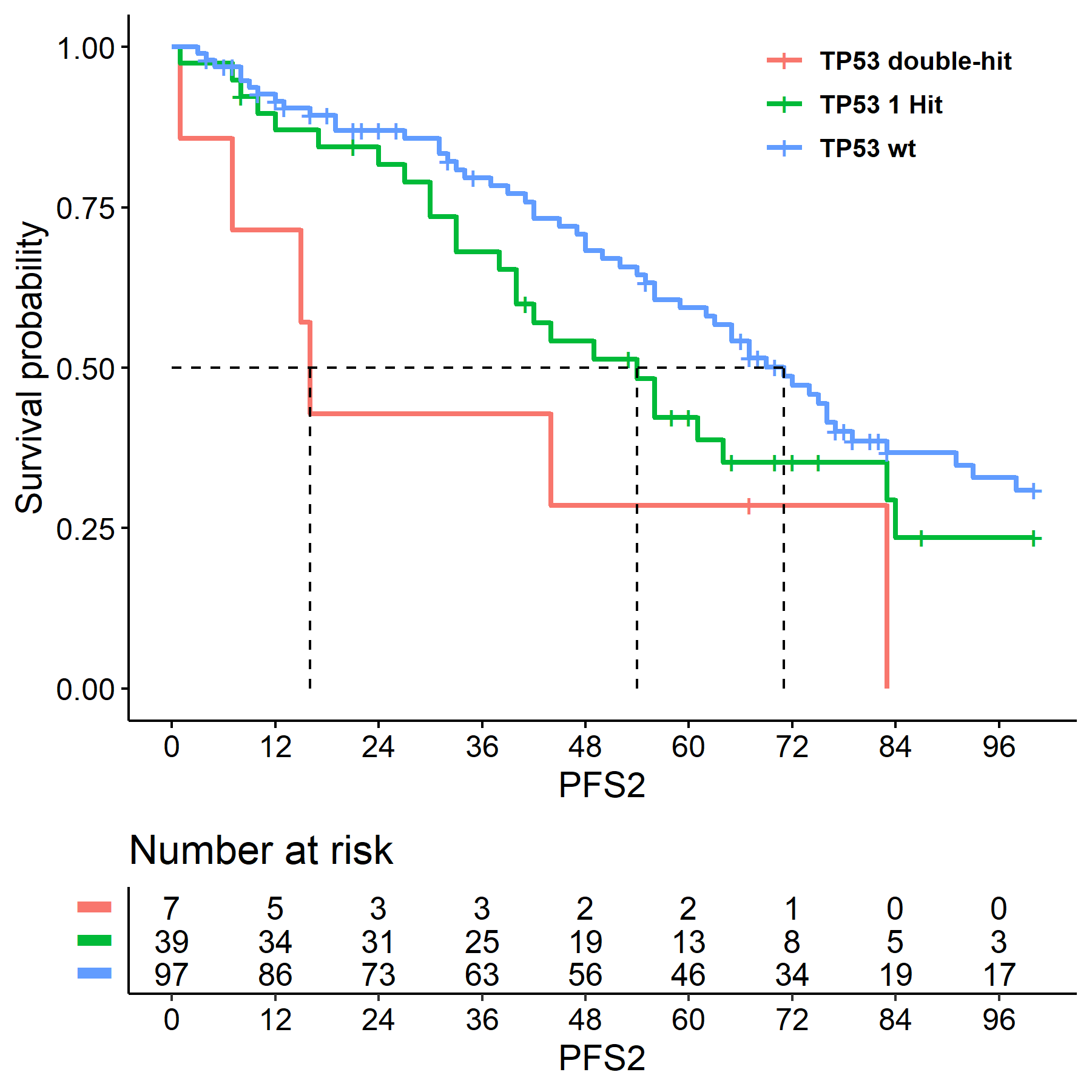


**
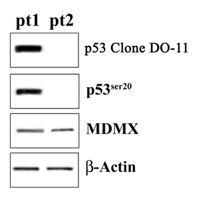
**

***Figure 2 SI: Effect of hetero- and homozygous deletion on p53 protein.*** Western blotting analysis on CD138^+^ cell derived from two MM patients, was performed as previously published^17^. Patient 1 (pt1) carrying one copy loss of *TP53* still conserved an intact activation of p53 pathway, whereas patient 2 (pt2) with double hit showed a complete inactivation of the same pathway. The primary antibodies used for Immunoblotting were purchased from Santa Cruz (anti-β-Actin) and Cell Signaling Technology® Inc (anti-p53ser20 and anti-MDMX). Dr Jean-Christophe Bourdon (University of Dundee/Ninewells hospital College of Medicine Jacqui Wood Cancer Centre, UK) kindly provided anti-p53 clone DO-11. The secondary antibodies used for Immunoblotting were purchased from GE Healthcare (anti-rabbit and anti-mouse) and Santa Cruz (anti-goat).

**
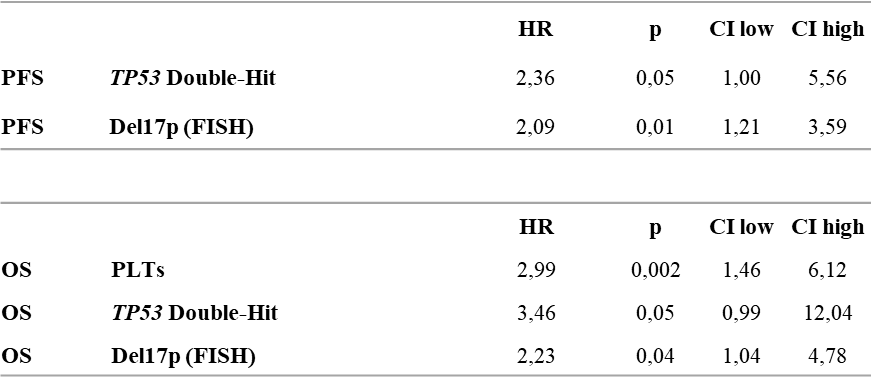
**

**Table 4 SI - Multivariable model analysis on PFS and OS**


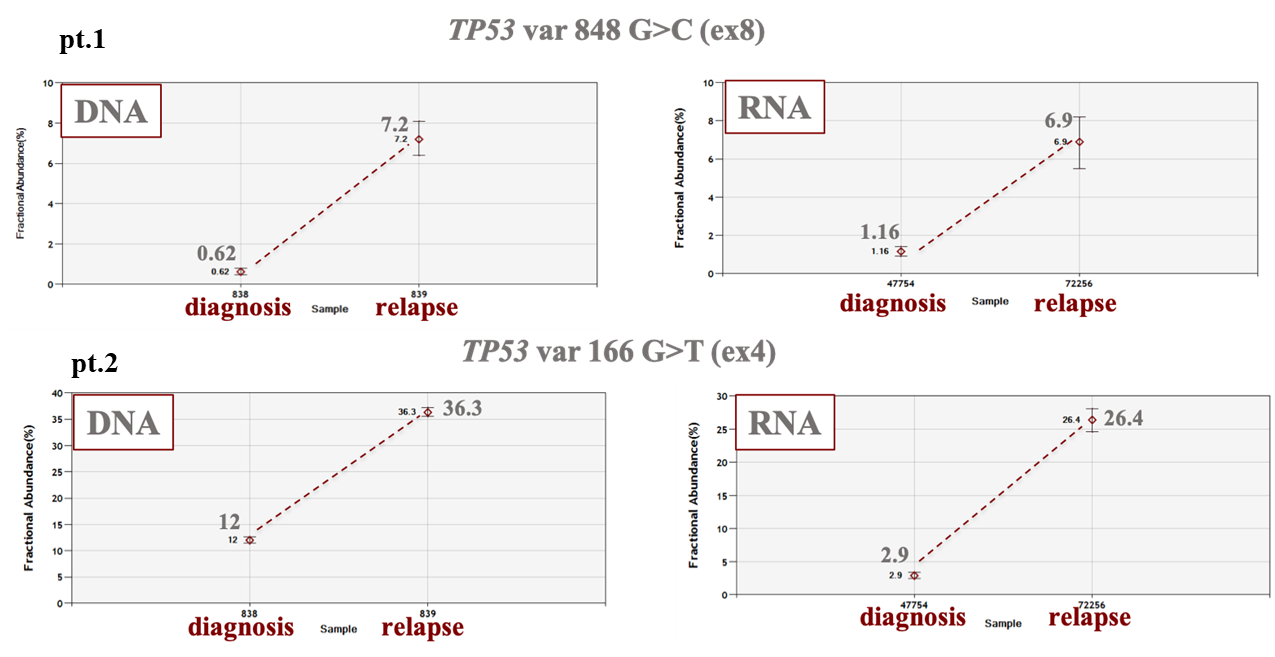


**Figure 3 SI: TP53 mutations validation by specific ddPCR assay.** In patient 1 (pt.1), the frequency of TP53 var 166 G>T (ex4) displays an increase from 12 to 36,3%, which is confirmed both at DNA as well as RNA level. In patients 2 (pt.2), the frequency of TP53 var 848 G>C (ex8) reported a variation from 0,62 to 7,2% and, even though this represent a slightly increase of frequency between diagnosis and relapse, we were able to confirm it both at DNA and at RNA level despite its strong sub-clonal level.
